# Association of interleukin 6 -174 G/C polymorphism with coronary artery disease and circulating IL-6 levels: a systematic review and meta-analysis

**DOI:** 10.1007/s00011-021-01505-7

**Published:** 2021-09-30

**Authors:** Himanshu Rai, Roisin Colleran, Salvatore Cassese, Michael Joner, Adnan Kastrati, Robert A. Byrne

**Affiliations:** 1 Cardiovascular Research Institute Dublin, Mater Private Network, Dublin, Ireland; 2 School of Pharmacy and Biomolecular Sciences, RCSI University of Medicine and Health Sciences, Dublin, Ireland; 3 Klinik für Herz- und Kreislauferkrankungen, Deutsches Herzzentrum München, Technische Universität München, Munich, Germany; 4 DZHK (German Centre for Cardiovascular Research), Partner Site Munich Heart Alliance, Munich, Germany

**Keywords:** Interleukin 6, Single nucleotide polymorphism, Coronary artery disease

## Abstract

**Introduction:**

Circulating IL-6 levels and at least one polymorphic form of *IL6* gene (*IL6* -174 G/C, rs1800795) have been shown to be independently associated with coronary artery disease (CAD) by several investigators. Despite more than 12 published meta-analyses on this subject, association of -174 G/C with CAD, especially amongst distinct ancestral population groups remain unclear. We, therefore, conducted a systematic review and an updated meta-analysis to comprehensively ascertain the association of *IL6* -174 G/C with CAD and circulating IL-6 levels.

**Materials and methods:**

Relevant case–control/cohort studies investigating association of -174 G/C with CAD and circulating IL-6 levels were identified following a comprehensive online search. Association status for CAD was determined for the pooled sample, as well as separately for major ancestral subgroups. Association status for circulating IL-6 levels was assessed for the pooled sample, as well as separately for CAD cases and CAD free controls. Study-level odds ratios (OR) and 95% confidence intervals (CI) were pooled using random/fixed-effects model.

**Results:**

Quantitative synthesis for the CAD endpoint was performed using 55 separate qualifying studies with a collective sample size of 51,213 (19,160 cases/32,053 controls). Pooled association of -174 G/C with CAD was found to be statistically significant through dominant (OR 1.15; 95% CI 1.05–1.25, *p* = 0.002) as well as allelic genetic model comparisons (OR 1.13, 95% CI 1.06–1.21, *p* = 0.0003). This effect was largely driven by Asian and Asian Indian ancestral subgroups, which also showed significant association with CAD in both genetic model comparisons (OR range 1.29–1.53, *p* value range ≤ 0.02). Other ancestral subgroups failed to show any meaningful association.

Circulating IL-6 levels were found to be significantly higher amongst the ‘C’ allele carriers in the pooled sample (Standard mean difference, SMD 0.11, 95% CI 0.01–0.22 pg/ml, *p* = 0.009) as well as in the CAD free control subgroup (SMD 0.10, 95% CI 0.02–0.17 pg/ml, *p* = 0.009), though not in the CAD case subgroup (SMD 0.17, 95% CI = − 0.02 to 0.37, *p* = 0.12).

**Conclusions:**

The present systematic review and meta-analysis demonstrate an overall association between *IL6* -174 G/C polymorphism and CAD, which seems to be mainly driven by Asian and Asian Indian ancestral subgroups. Upregulation of plasma IL-6 levels in the ‘C’ allele carriers seems to be at least partly responsible for this observed association. This warrants further investigations with large, structured case–control studies especially amongst Asian and Asian Indian ancestral groups.

**Supplementary Information:**

The online version contains supplementary material available at 10.1007/s00011-021-01505-7.

## Introduction

Interleukin 6 (IL-6) is a circulating bioactive peptide of 23.7 kDa and acts as both a pro-inflammatory cytokine and an anti-inflammatory myokine. This endogenous pyrogen primarily originates from mononuclear phagocytes but also, in part, from fibroblasts, T and B lymphocytes and vascular endothelial cells [1, 2]. It functions in inflammation and maturation of B cells [3], and is encoded by *IL6* gene (located at chromosome 7p21–14) which is known to have several polymorphic variants [4]. Circulating IL-6 levels and at least one polymorphic form of *IL6* gene have been reported to be independently associated with coronary artery disease (CAD), at least amongst Caucasians [5, 6].

G to C substitutions at -174 bp (-174 G/C; rs1800795) and -572 bp (-572 G/C, rs1800796) in the promoter region of *IL6* gene are the most important single nucleotide polymorphisms (SNPs) with respect to CAD [5–7]. These two SNPs have been reported to be functional, seem to have a co-operative influence over each other and are capable of altering circulating IL-6 levels through complex interactions depending on the haplotype [4, 8]. There have been at least 12 published meta-analyses investigating the association of common *IL6* SNPs with CAD, myocardial infarction (MI; or surrogates), and related clinical presentations. [5–7, 9–17] However, flawed ethnic stratifications in these meta-analyses yielded inconsistent results [5–7, 9–17]. Against this background, our objectives in this systematic review and meta-analysis were to: (i) ascertain the overall association of *IL6 *-174 G/C polymorphism with CAD, as well as separately amongst different ancestral populations, (ii) investigate the association of this polymorphic form with circulating IL-6 levels.

## Material and methods

Relevant guidelines in the HuGE Review Handbook, version 1.0 [18] as well as the PRISMA (Preferred Reporting Items for Systematic Reviews and Meta-Analyses) statement [19] were strictly adhered to while undertaking the present systematic review and meta-analysis.

### Search strategy and study selection criteria

We systematically searched the databases of the US National Institutes of Health (PubMed), EMBASE, MEDLINE, Scopus and Web of Knowledge for relevant articles published online until May 2021. Specific search headings as well as open text fields were used for the online publication search. Reference lists of relevant published meta-analyses were also scanned for identifying additional articles. Combination of broad search headings such as ‘interleukin 6’ OR ‘*IL6*’ OR rs1800795 (dbSNP ID’s or rs number) paired with ‘coronary artery disease’ OR ‘CAD’ OR ‘myocardial infarction’ OR ‘MI’ OR ‘acute coronary syndrome’ OR ‘ACS’ AND ‘polymorphism’ OR ‘mutation’ OR ‘single nucleotide polymorphism’ OR ‘SNP’ were used for online search. Our search was limited to publications in the English language and restricted to articles relating to humans.

Hierarchical model for study selection was used: initially the study title was assessed for relevance, followed by the abstract and finally, the full text. To qualify for inclusion, the relevant study had to be either a case–control study or cohort study with a well-documented CAD case group (diagnosed CAD, MI, ACS, unstable/stable angina pectoris) compared against a CAD free control group. To be included, all studies had to satisfy the following criteria: (1) original, published in a peer-reviewed journal, and available online, (2) case–control or cohort design, (3) providing complete genotypic data crucial for calculation of odds ratios (OR), confidence intervals (CIs) and p-values, (4) CAD diagnosis amongst cases had to be based on angiographic or electrocardiographic assessment whilst controls had to be free of any history or evidence of CAD, (5) published in the English language with online accessibility, and (6) genotype frequencies amongst controls satisfying Hardy–Weinberg equilibrium (HWE). We assessed departure from HWE amongst controls in each study using the goodness-of-fit × 2 test. Incidences of non-conformation to HWE approximation (*p* < 0.05) resulted in the exclusion of the study. Conference abstracts and case reports/studies not providing adequate information were also excluded. Publications lacking enough data to generate both dominant and allelic genetic models were identified and their corresponding authors were formally requested for supplying missing data via three periodic emails, spaced 1 week apart. We included the study after receiving the complete data. In case all efforts to retrieve the missing data failed, we included studies where enough data was available to construct at least one genetic model. If relevant data was not made available even after three consecutive requests and published data was not sufficient to construct even one genetic model, the study in question was excluded. Further study selection to ascertain the association of *-*174 G/C polymorphism with circulating IL-6 levels was done from the already searched publications.

### Data collection and quality assessment

Raw data were transcribed from selected publications on Microsoft-Excel worksheets where further calculations were performed. Studies qualifying for testing association of *-*174 G/C with CAD were stratified into ancestral subgroups such as European ancestry, Middle Eastern ancestry, Asian ancestry, Asian Indian ancestry, African ancestry and Mixed ancestry. Studies were categorized based on the ancestral background of the majority of the studied population. Studies were stratified into ‘CAD cases’ and ‘CAD free control’ subgroups while testing for an association of *-*174 G/C polymorphism with circulating IL-6 levels.

Quality assessment of the included studies was performed using Newcastle–Ottawa scale (NOS) (http://www.ohri.ca/programs/clinical_epidemiology/oxford.asp). The NOS is a star-based rating system where a study with a full score can earn 9 stars. A NOS rating of 5–9 stars was indicative of a good quality study, while a score of 0–4 stars indicated a poor quality study [20]. The NOS rating tool involves evaluation of (a) selection methods of study participants, (b) comparability amongst cases and control groups, and (c) exposure and outcome. Included studies were independently assessed for quality by both authors; disagreements were then resolved by consensus.

Environmental factors have been known to have a profound impact on the association profiles of genetic polymorphisms. Since none of our included studies provided raw calculable data on environmental factors, we were unable to test the impact of this relationship.

### Statistical techniques

Calculations were carried out using windows based RevMan version 5.3.5 (The Cochrane Collaboration, 2014) and SPSS version 25 (IBM^®^ corporation).

#### Summary effect measures

Odds ratios (ORs) were calculated using bivariate, random (DerSimonian–Laird method) [21] or fixed-effect model (Mantel–Haenszel method) [22]. Summary ORs and their 95% confidence intervals (CIs) were calculated separately for dominant and allelic genetic models. Analytic models (random or fixed) were chosen based on observed heterogeneity within the group/subgroup. The calculated OR and 95% CI for each study revealed the level of association (if any). The pooled OR was estimated from individual study ORs employing a *Z* test. The summary effect measure for the association of -174 G/C with circulating IL-6 levels was pooled standard mean difference (SMD) with its 95%CI (in pg/ml). SMDs were estimated for each study after which a *Z* test was employed to ascertain a pooled SMD. For both summary effect measures, a pooled *p* value of < 0.05 indicated statistical significance and the corresponding *Z* value indicated the level of association.

#### Heterogeneity assessment

Existence of heterogeneity was tested using a *Q* test. Resulting Higgins *I*^2^ statistics (*I*^2^) and Cochrane’s *Q* statistics (*P*_Q_) for each study group/subgroup indicated inherent heterogeneity. A heterogeneous group/subgroup was assumed to be with a resultant *P*_Q_ cut-off < 0.01 [23]. The *I*^2^ value cut offs of 25%, 50% and 75% indicated low, moderate and high heterogeneity, respectively [24]. Random effects for calculation of summary effect measures were used if the group/subgroup yielded a *P*_Q_ value of ≤ 0.01 coupled with an *I*^2^ value of ≥ 50%. Conversely, fixed effect was used for summary effect measure estimation if the group/subgroup yielded a *P*_Q_ value of > 0.01 coupled with an *I*^2^ value of < 50%. Subgroup differences were also assessed assuming similar *P*_Q_ and *I*^2^ cut-offs.

#### Detection of publication bias

We employed two of the most accepted statistical tools for publication bias detection in the present meta-analysis. Publication bias in each group of ≥ 3 studies was visually detected using Begg’s funnel plot [25], while the statistical estimates for each group/subgroup were calculated using Egger’s test.[26] An Egger’s *p* value of < 0.05 was considered statistically significant and indicated the possible existence of publication bias in the group/subgroup in question.

#### Sensitivity analysis

Sensitivity analysis was performed separately in each study group/subgroup (with ≥ 5 studies). We repeated the analysis after the omission of one study after another in each qualifying group/subgroup. This exercise was performed to see if the results in any group/subgroup altered substantially, i.e. a change from non-association to a significant association or the other way around. Absence of such alteration in results indicates the robustness of the meta-analysis in question.

## Results

**Fig. 1:**
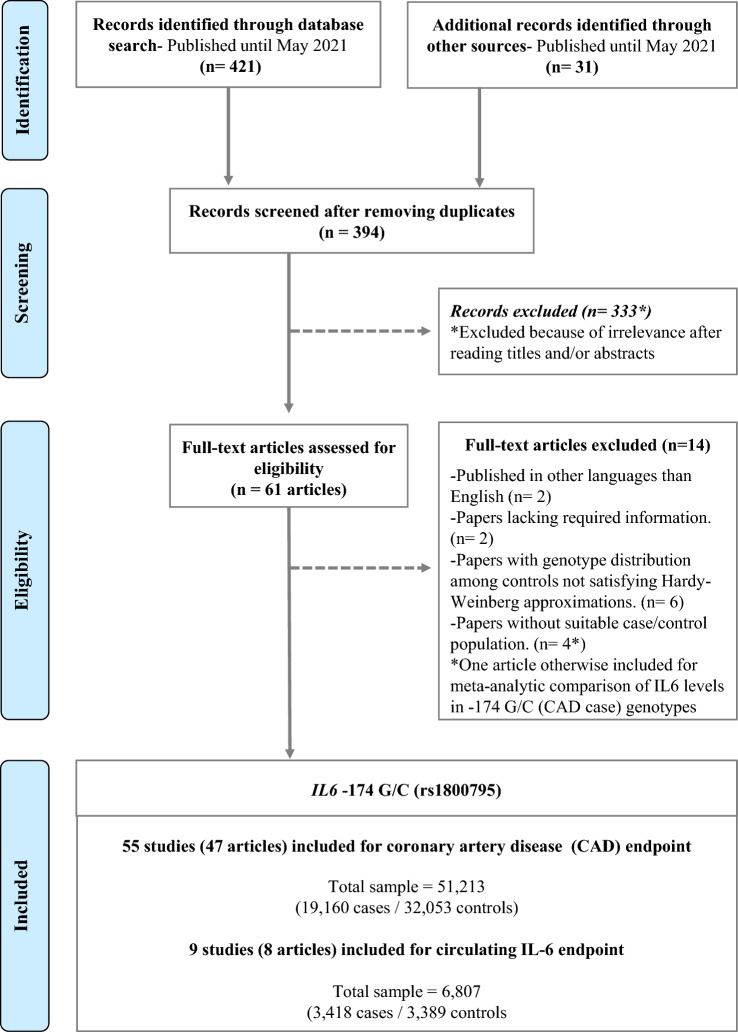
Study selection flowchart

Screening of 394 records led to the identification of 47 relevant articles. A total of 55 different studies (extracted from 47 articles) were included to test the association of -174 G/C with CAD [27–73]. The study selection process is explained in detail in Fig. 1. Table 1 contains complete details of all included studies for the CAD endpoint, while Table 2 lists studies included for circulating IL-6 levels endpoint. Sample assessed and inherent heterogeneity of studied groups and subgroups included for CAD endpoint are shown in Supplementary Table 1. Meta-analysis results obtained for CAD endpoint are summarized in Supplementary Table 2.

**Table 1:** List of studies included for CAD endpoint

Study	Year	Country/region	Sample assessed (cases/controls)	Genotypic distribution in cases (CC:GC:GG)	Genotypic distribution in controls (CC:GC:GG)	MAF (cases/controls)	Outcome	Newcastle–Ottawa scale rating
*European ancestry*								
Humphries et al*. *[27]	2001	UK	160/2560	25:95:40	470:1263:827	45.3/43.0	CAD	7/9 stars
Georges et al. (1a) [28]	2001	UK (Belfast)	186/172	32:109:45	28:97:47	46.5/44.5	MI	8/9 stars
Georges et al. (1b) [28]	2001	France	428/500	72:231:125	77:239:184	43.8/39.3	MI	8/9 stars
Basso et al. [29]	2002	UK	498/1109	78:259:161	185:549:375	41.7/41.4	CAD	8/9 stars
Nauck et al. (a) [30]	2002	Germany	2575/729	499:1238:838	144:355:230	43.4/44.1	CAD	7/9 stars
Nauck et al. (b) [30]	2002	Germany	1365/729	261:668:436	144: 355: 230	43.6/44.1	MI	7/9 stars
Bennet et al. [31]	2003	Sweden	1157/1500	275:577:305	348:754:398	48.7/48.3	MI	8/9 stars
Georges et al. (2) [32]	2003	Germany	844/311	154:431:259	56:168:87	43.8/45.0	CAD	7/9 stars
Kelberman et al. (a) [33]	2004	North Europe	229/244	40:100:89	53:120:71	39.3/46.3	MI	6/9 stars
Kelberman et al. (b) [33]	2004	South Europe	278/317	21:119:138	28:120:169	29.0/27.8	MI	6/9 stars
Licastro et al. [34]	2004	Italy	138/97	15:88:35	7:44:46	42.8/29.9	MI	7/9 stars
Lieb et al. (a) [35]	2004	Germany (Rosenberg)	743/1023	141:362:240	193:499:331	43.3/43.3	MI	8/9 stars
Lieb et al. (b) [35]	2004	Germany (Augsburg)	579/1023	103:265:211	193:499:331	40.7/43.3	MI	8/9 stars
Chiapelli et al. (a) [36]	2005	Northern Italy	138/204	15:88:35	22:81:101	42.8/30.6	AMI	8/9 stars
Chiapelli et al. (b) [36]	2005	Southern Italy	66/53	6:24:36	2:25:26	27.3/27.4	AMI	8/9 stars
Densem et al. [37]	2005	UK	116/519	32:53:31	88:229:202	50.4/39.0	CAD	6/9 stars
Rosner et al. [38]	2005	USA	522/2089	85:233:204	294:973:822	38.6/37.4	MI	7/9 stars
Seifart et al. [39]	2005	Germany	112/243	12:51:49	46:107:90	33.5/40.9	CAD	8/9 stars
Sie et al. (a) [40]	2006	Netherlands	463/5221	83:222:158	882:2451:1888	41.9/40.4	CAD	7/9 stars
Sie et al. (b) [40]	2006	Netherlands	208/5476	38:97:73	927:2576:1973	41.6/40.4	MI	7/9 stars
Sarecka et al. [41]	2008	Poland	178/202	42:93:43	37:105:60	49.7/44.3	CAD	7/9 stars
Sarecka-Hujjar et al. [42]	2008	Poland	177/202	42:92:43	37:105:60	49.7/44.3	CAD	7/9 stars
Aker et al. [43]	2009	Germany	218/245	27 (CC):191 (GC + GG)	42 (CC): 203 (GC + GG)	NA	CAD	6/9 stars
Berg et al. [44]	2009	Norway	130/130	43 (CC): 87 (GC + GG)	19 (CC): 81(CG + GG)	NA	CAD	8/9 stars
Rios et al. (a) [45]	2010	Brazil (Caucasian-Brazilians)	276/138	28:90:158	10:46:82	26.4/23.9	CAD	7/9 stars
Bennermo et al. [46]	2011	Sweden	356/378	87:150:119	93:176:109	45.5/47.9	MI	7/9 stars
Lima-Neto et al. [47]	2013	Brazil	102/108	NA	NA	37.2/33.8	MI	6/9 stars
Hatzis et al. [48]	2014	Greece	347/299	55:147:145	39:129:131	37.0/34.6	CAD	8/9 stars
Mitrokhin et al. [69]	2017	Russia	198/116	36:100:62	26:58:32	43.4/47.4	CAD	5/9 stars
*Middle Eastern ancestry*								
Tutun et al. [49]	2006	Turkey	21/50	4:6:11	0:15:35	33.3/15.0	CAD	8/9 stars
Sekuri et al. [50]	2007	Turkey	115/105	5:49:61	7:41:57	25.7/26.2	Premature CAD	6/9 stars
Ghazouani et al. [51]	2010	Tunisia	418/406	10:110:298	7:102:297	15.6/14.3	CAD	8/9 stars
Coker et al. [52]	2011	Turkey	167/235	9:56:102	13:81:141	22.2/22.8	MI	7/9 stars
Jabir et al. [53]	2016	Saudi Arabia	90/89	3:25:62	3:23:63	17.2/16.3	CAD	5/9 stars
*Asian ancestry*								
Kuo et al. [54]	2008	China	58/77	4:27:27	13:32:32	30.2/37.7	CAD	7/9 stars
Fan et al. [55]	2011	China	84/130	0:0:84	0:1:129	0.0/0.03	CAD	6/9 stars
Li et al. [56]	2015	China	365/365	39:113:213	15:105:245	26.2/18.5	CAD	7/9 stars
Wang et al. [57]	2015	China	402/402	78:171:153	51:169:182	40.7/33.7	CAD	7/9 stars
Yang et al. [58]	2015	China	410/410	49:163:198	25:146:239	31.8/23.9	CAD	7/9 stars
Hongmei et al. [59]	2016	China	275/296	0:19:256	0:14:282	3.5/2.4	CAD	6/9 stars
Chen et al. [72]	2018	China	429/350	56:218:155	27:133:190	28.5/26.7	CAD	7/9 stars
*Asian Indian ancestry*								
Banerjee et al. [60]	2009	India	210/232	8:43:159	4:57:171	14.1/14.0	CAD	7/9 stars
Babu et al. [61]	2012	India	651/432	223:294:134	91:206:135	56.8/44.9	ACS	6/9 stars
Bhanushali and Das[62]	2013	India	100/150	3:20:77	4:25:121	13.0/11.0	CAD	7/9 stars
Mishra et al. (a) [63]	2013	India (Primary cohort)	310/230	9:83:218	4:54:172	16.3/13.5	CAD	6/9 stars
Mishra et al. (b) [63]	2013	India (Replication cohort)	290/230	4:82:204	4:54:172	15.5/13.5	CAD	6/9 stars
Phulukdaree et al. [64]	2013	South Africa	41/100	1:11:29	10:32:58	15.9/26.0	CAD	5/9 stars
Satti et al. [65]	2013	Pakistan	36/52	7:11:18	0:14:38	34.7/13.5	CAD	5/9 stars
Biswas et al. [66]	2014	India	500/500	13:139:348	1:92:407	16.5/9.4	MI	7/9 stars
Galimudi et al. [67]	2014	India	200/200	26:102:72	18:69:113	38.5/26.3	CAD	7/9 stars
Ansari et al. [68]	2016	Pakistan	340/310	13:85:242	3:71:236	16.3/12.4	CAD	7/9 stars
Mastana et al. [70]	2017	India	138/131	1:32:105	1:39:91	12.3/15.6	CAD	6/9 stars
Shabana et al. [71]	2018	Pakistan	426/219	99:133:194	33:90:96	38.8/35.6	CAD	5/9 stars
*African ancestry*								
Rios et al. (b) [45]	2010	Brazil (African-Brazilians)	138/115	6:36:96	3:43:69	17.4/21.3	CAD	7/9 stars
*Mixed ancestry*								
Almeida et al. [73]	2019	Mexico	159/300	6:19:134	08:85:207	9.7/16.8	CAD	8/9 stars

*IL6* interleukin 6 gene, *MAF* minor allele frequency, *CAD* coronary artery disease, *MI* myocardial infarction, *AMI* acute myocardial infarction, *ACS* acute coronary syndrome

**Table 2:** List of studies included for circulating IL-6 endpoint

Studies	Year	Ancestry	Sample size [‘C’ allele carriers (CC + GC)/wild type genotype (GG)]	IL-6 levels (pg/ml) in ‘C’ allele carriers (CC and GC)	IL-6 levels (pg/ml) in wild type genotype (GG)
*CAD cases*					
Basso et al. [29]	2002	European	117/60	2:95 ± 3.67	2.99 ± 3.23
Bennet et al. [31]	2003	European	852/295	1.59 ± 2.90	1.73 ± 3.40
Kelberman et al.(a) [33]	2004	European	117/76	1.67 ± 1.11	1.83 ± 1.22
Kelberman et al.(b) [33]	2004	European	78/87	2.22 ± 1.48	2.20 ± 1.47
Lieb et al. [35]	2004	European	476/211	2.12 ± 9.50	2.00 ± 8.43
Bennermo et al. [46]	2011	European	237/119	0.79 ± 1.72	0.81 ± 1.87
Satti et al. [65]	2013	Asian Indian	18/18	51.55 ± 17.65	27.00 ± 4.00
Biswas et al. [66]	2014	Asian Indian	152/348	28.09 ± 108.71	12.60 ± 33.54
Toutouzas et al. [74]	2017	European	127/30	4.71 ± 2.29	2.71 ± 1.08
*CAD free controls*					
Basso et al. [29]	2002	European	183/98	2.63 ± 3.13	2.66 ± 2.68
Bennet et al. [31]	2003	European	1102/398	1.29 ± 0.72	0.93 ± 2.25
Kelberman et al. (North Europe) [33]	2004	European	156/62	1.29 ± 0.72	1.20 ± 0.67
Kelberman et al. (South Europe) [33]	2004	European	95/103	1.28 ± 0.85	1.14 ± 0.75
Bennermo et al. [46]	2011	European	269/109	0.66 ± 0.91	0.58 ± 0.65
Biswas et al. [66]	2014	Asian Indian	407/407	10.1 ± 55.63	7.28 ± 6.71

### Role of -174 G/C polymorphism in CAD

**Fig. 2:**
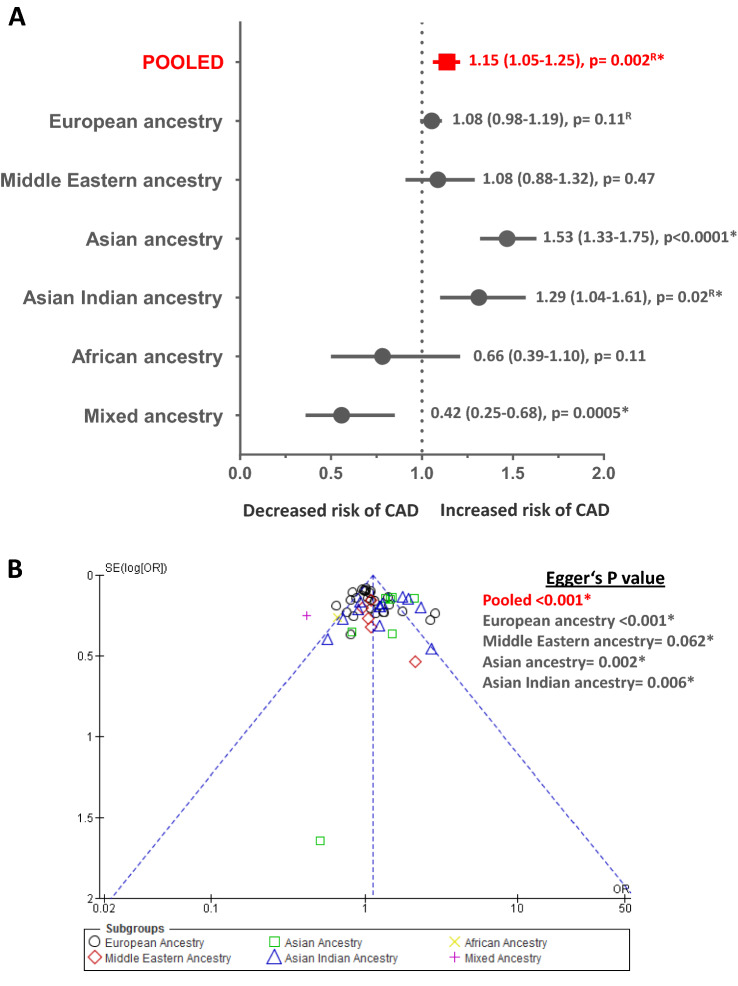
Meta-analysis results and publication bias assessment employing dominant genetic model comparisons (CC + GC versus GG) for CAD endpoint. Panel **A** Condensed Forest plot depicting associations of *IL6* -174 G/C polymorphism with CAD. ^R^ Effect sizes for “Pooled” as well as for European and Asian Indian ancestral subgroups displaying high level of heterogeneity were estimated using random effects for analysis. Effect sizes for Middle Eastern, Asian, African and Mixed ancestral subgroups were estimated using fixed effects. Effect sized are displayed as Odds Ratio (95% Confidence Interval). *Statistically significant *p* value of < 0.05. Panel **B** Publication bias assessment using Begg’s funnel plot with Egger’s estimates amongst the group of studies investigating the role *IL6* -174 G/C polymorphism in CAD. Each point in this figure represents the odds ratio (OR) obtained for a study plotted against its standard error (SE). Different indicators have been used for studies belonging to each ancestral subgroup. *Statistically significant *p* value of < 0.05

**Fig. 3:**
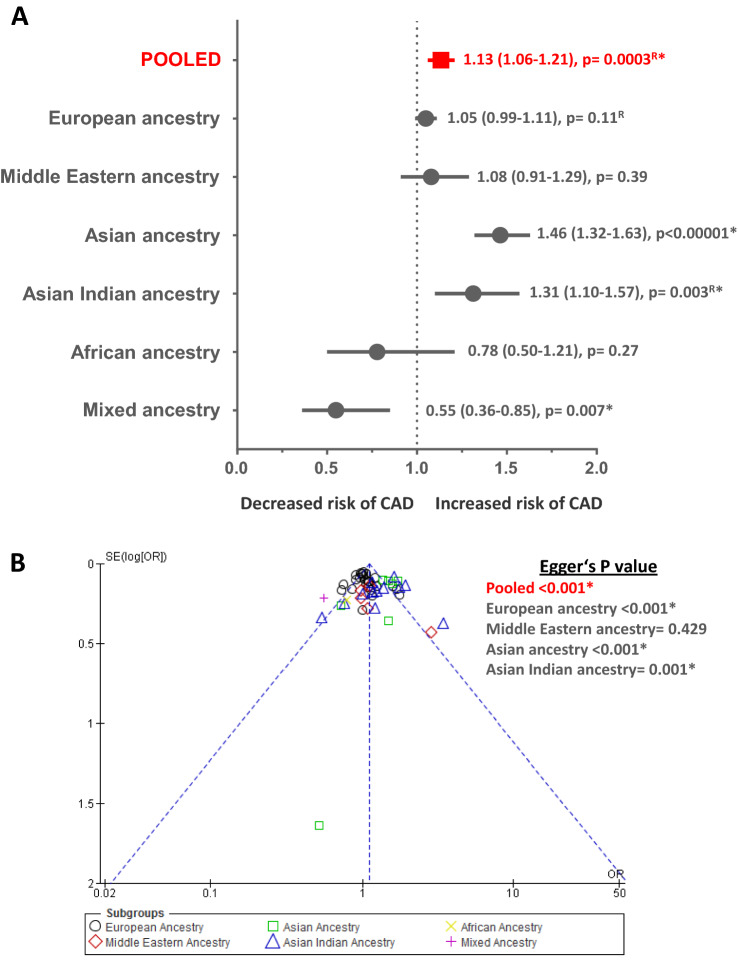
Meta-analysis results and publication bias assessment employing allelic genetic model comparisons (Allele C versus Allele G) for CAD endpoint. Panel **A** Condensed Forest plot depicting associations of *IL6* -174 G/C polymorphism with CAD. ^R^ Effect sizes for “Pooled” as well as for European and Asian Indian ancestral subgroups displaying a high level of heterogeneity were estimated using random effects for analysis. Effect sizes for Middle Eastern, Asian, African and Mixed ancestral subgroups were estimated using fixed effects. Effect sized are displayed as Odds Ratio (95% Confidence Interval). *Statistically significant *p* value of < 0.05. Panel **B** Publication bias assessment using Begg’s funnel plot with Egger’s estimates amongst the group of studies investigating the role *IL6* -174 G/C polymorphism in CAD. Each point in this figure represents the odds ratio (OR) obtained for a study plotted against its standard error (SE). Different indicators have been used for studies belonging to each ancestral subgroup. *Statistically significant *p* value of < 0.05

A total of 55 case–control/cohort genetic association studies on *-*174 G/C, with a total sample of 51,213 (19,160 cases/32,053 controls) were analyzed [27–73]. The pooled group showed significant heterogeneity as the included studies belonged to 6 distinct ancestral subgroups. (Supplementary Table 1) Our pooled results via both genetic models were obtained using random effects. Pooled comparisons using dominant (CC + GC vs. GG) and allelic genetic models (allele C vs. allele G) suggested statistically significant association with CAD [number of studies (*n*_st_) = 52, OR 1.15, 95% CI 1.05–1.25, *Z* = 3.03 and *p* = 0.002 and *n*_st_ = 53, OR 1.13, 95% CI 1.06–1.21, *Z* = 3.65 and *p* = 0.0003, respectively]. (Fig. 2, Panel A and Fig. 3, Panel A, respectively, for dominant and allelic model, Supplementary Figs. 1 and 2, respectively, for dominant and allelic model and Supplementary Table 2).

Similarly, summary effect measures for all ancestral subgroups, respectively, were also obtained using appropriate effects for analysis. Results obtained for Asian ancestry [54–59, 72] and Asian Indian ancestry [60–68, 70, 71] subgroups displayed significant positive association via both genetic models (*n*_st_ = 7, OR range 1.46–1.53, *Z* value range 6.04–7.03, *p* value range =  < 0.0001 to < 0.00001 and *n*_st_ = 12, OR range 1.29–1.31, *Z* value range = 2.28–2.95, *p* value range = 0.02–0.003, respectively). On the other hand, Mixed ancestry subgroup [73] showed a negative association with CAD (*n*_st_ = 1, OR range 0.42–0.55, *Z* value range = 2.71–3.50, *p* value range = 0.007–0.0005). Other ancestral subgroups such as European ancestry [27–48, 69], Middle Eastern ancestry [49–53] and African ancestry [45] did not seem to be associated with CAD (*p *value range = 0.11–0.47). (Fig. 2, Panel A and Fig. 3, Panel A, respectively, for dominant and allelic model, Supplementary Figs. 1 and 2, respectively, for dominant and allelic model and Supplementary Table 2) Understandably, substantial subgroup differences were observed (*I*^2^ range = 82.5–84.1% and *P*_Q_ range = <0.0001 to <0.00001) (Supplementary Table 2).

### Association of -174 G/C polymorphism with circulating IL-6 levels

**Fig. 4:**
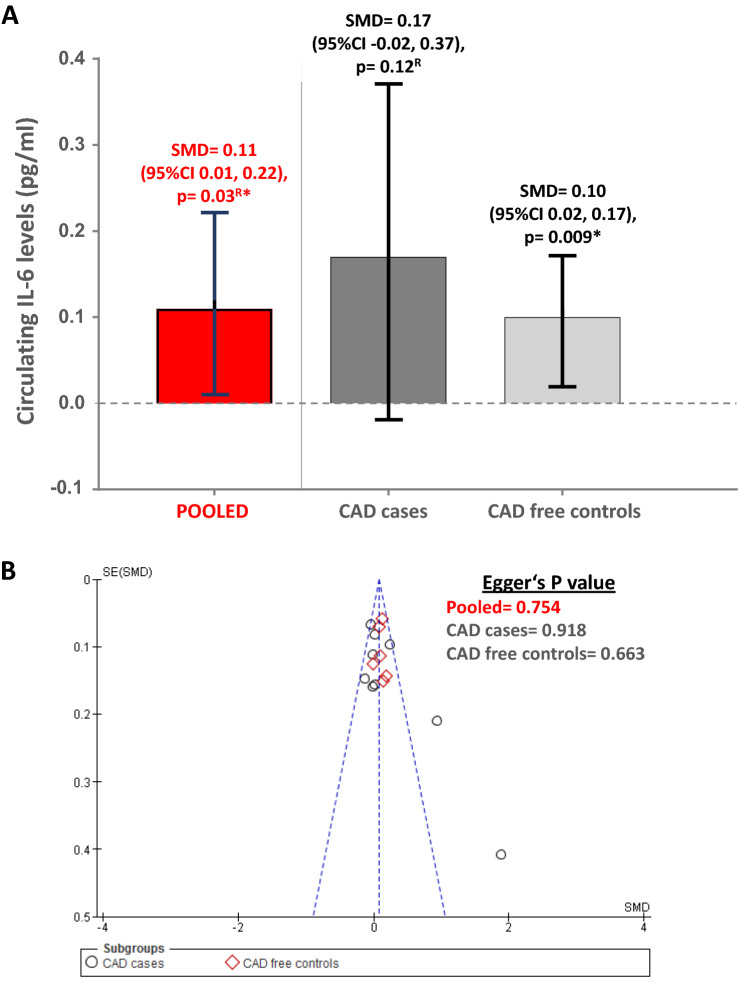
Meta-analysis results depicting differences in circulating IL-6 levels amongst ‘C’ allele carriers versus GG homozygotes as well as publication bias assessment results in the included groups/subgroups. Panel **A** Comparison of IL-6 levels between ‘C’ allele carriers as compared to the rest (CC+GC vs. GG) separately amongst CAD cases and CAD free controls. ^R^Standard mean difference for “Pooled” as well as CAD case subgroup were estimated using random effects owing to high levels of inherent heterogeneity. Standard mean difference for CAD free control subgroup which displayed low levels of inherent heterogeneity were estimated using fixed effects. Standard mean difference and its 95% Confidence Interval is depicted in the bar charts. *Statistically significant *p* value of < 0.05. Panel **B** Begg’s funnel plot with Egger’s estimates was obtained for comparison of circulating IL-6 levels between ‘C’ allele carries as compared to the rest (CC + GC vs. GG). *Statistically significant *p* value of < 0.05. Each point in each figure represents the standard mean difference (SMD, in pg/ml) obtained for a study plotted against its standard error (SE). Different indicators have been used for studies belonging to ‘CAD cases’ and ‘CAD free controls’ subgroups

IL-6 levels amongst ‘C’ allele carriers were compared against the rest in the overall sample and separately amongst CAD cases (*n*_st_ = 9; 3418 subjects) [29, 31, 33, 35, 46, 65, 66, 74] and CAD free controls (*n*_st_ = 6; 3389 subjects) [29, 31, 33, 46, 66]. ‘C’ allele carriers in the overall sample were associated with significantly higher plasma levels of IL-6 (SMD 0.11, 95% CI 0.01–0.22 pg/ml, *Z* value = 2.14, *p* = 0.03). While CAD case subgroup did not yield significant association (*p* = 0.12), IL-6 levels were found to be significantly higher amongst the ‘C’ allele carriers in the CAD free control subgroup (SMD 0.10, 95% CI 0.02–0.17 pg/ml, *Z* value = 2.62, *p* = 0.009) as shown in Fig. 4, Panel A and Supplementary Fig. 3. No evidence of publication bias was seen in pooled group as well as in the subgroups, which validated the derived associations (Egger’s *p* value range = 0.663–0.918) (Fig. 4, Panel B).

### Publication bias assessment and sensitivity analysis

Each group or subgroup with ≥ 3 included studies was assessed for existing publication bias using Begg’s funnel plot test [25] and Egger’s test [26]. Begg’s funnel plots and Egger’s *p* values for each qualifying group/subgroup constructed for -174 G/C for CAD endpoint are displayed in Fig. 2, Panel B and Fig. 3, Panel B (respectively, for dominant and allelic model), while for circulating IL-6 levels endpoint in Fig. 4, Panel B. Each point in these plots represents the OR or SMD obtained for an included study plotted against its standard error (SE). Different indicators have been used for studies belonging to different ancestral subgroups/CAD cases or CAD free control subgroups. All these points seem to be generally contained within the inverted cone, indicating limited existence publication bias. Egger’s *p* values seem to reach statistical significance for most of the ancestral groups and subgroups which could have been a direct result of inherent heterogeneity. This indicates that the use of ancestral stratification was also not sufficient to tone down the possible existence of bias. On the other hand, we found no evidence of publication bias in subgroups constructed for circulating IL-6 endpoint.

Sensitivity analysis was performed in each study group/subgroup with ≥ 5 included studies. Studies were excluded one after another in these groups/subgroups and the analysis was repeated after each omission. We observed no instance of significant alteration from the original results, i.e. from lack of association to significant association or the other way around for both endpoints, which is an indicator of the robustness of the meta-analysis in question (Data not shown).

## Discussion

We present the most comprehensive and structured meta-analysis on the association between *IL6* -174 G/C polymorphism with CAD as well as circulating IL-6 levels. The main findings were: (i) pooled results indicated a significant association of -174 G/C polymorphism with CAD; however, the effect was driven by studies with participants belonging to Asian and Asian Indian ancestries; (ii) other major ancestries, including European and Middle Eastern displayed no evidence of such association; (iii) ‘C allele’ carriers, at least amongst CAD free controls seem to have significantly higher levels of circulating IL-6, which in part explains the association of this SNP with CAD.

Results obtained in our meta-analysis for the CAD endpoint are much more robust than the recent one on this subject [17], which incidentally also lacks ancestral stratification needed to identify drivers of seen association. The latest meta-analysis which is comparable to ours’ was from Hou and coworkers published in 2015 [6], Our results for -174 G/C polymorphism with CAD represents a complete shift from their results. First, our pooled results displayed overwhelmingly strong associations with CAD (*p* ≤ 0.0005, for both genetic models), in contrast to a milder level of associations (*p* = 0.01 in both models) reported by Hou et al. [6] Second, we tried to correctly stratify different ancestral populations into appropriate subgroups thus revealing a clear picture, which is in contrast to Hou et al. [6], who clubbed Europeans along with Indian, Turkish, Tunisian and Pakistani populations in their ‘Caucasian’ subgroup.

At least 4 promoter polymorphisms of the *IL6* gene at positions 597, 572, 373 along with 174 bp, have been known to influence *IL6* transcription through complex interactions determined by the haplotype [4]. We hypothesize that ‘C’ allele carriers in -174 G/C, through a variety of mechanisms, are more likely to have upregulated transcription and translation of *IL6* gene; are, therefore, associated with higher plasma concentrations of circulating IL-6, thus making them more susceptible to the development of atherosclerotic disease. We tested this hypothesis and found that while possibly the influence of concomitant medications [75], prevented the CAD case subgroup to yield significant association (*p* = 0.12), our CAD free control subgroup showed a clear association of ‘C’ allele carriers with elevated circulating IL-6 levels (*p* = 0.009).

This difference has also been observed locally at the transcriptional level where *IL6* mRNA expression is 10–40 fold higher in atherosclerotic as compared to healthy arteries [76]. IL-6 not only has a direct association with CAD; it also indirectly contributes to the development of atherosclerotic disease in several ways. Circulating IL-6 has been known to regulate fibrinogen—an acute-phase protein which is recognized as an important risk factor for atherosclerotic and thrombotic diseases [76]. It has also been reported to stimulate the differentiation of monocytes to macrophages, which contributes towards the growth of atherosclerotic plaques [77]. The effect of individual *IL6* gene SNPs on the regulation of plasma IL-6 levels have also been investigated before [4]. Since, at least 4 adjacent *IL6* polymorphic sites (-174 G/C, -373 A/T, -572 G/C and -597 G/A) have complex interactions between each of their transcriptional machineries [4], it this not easy to determine the effects of a single variant. *IL6* promoter haplotypes have been reported to be better predictors of transcription levels of *IL6* gene [4]. Investigating the synergistic effect of these possible haplotypes on CAD, MI or circulating IL-6 levels was not possible due to the lack of relevant published haplotypic data.

### Limitations

Meta-analyses on genetic association studies tend to have significant limitations. First, for some ancestral subgroups—only a few published reports with moderate sample sizes were available; their meta-analysis results should thus be interpreted with caution. More studies from these ancestral groups are warranted to establish these derived associations. Second, the fact that meta-analyses of association studies cannot inspect interference of linkage disequilibrium, it constitutes as a major limitation. Third, the presence of selection bias in individual included studies and the presence of publication bias in a meta-analysis of non-randomized, genetic association studies easily qualify to be the most important limitation. Several statistical tools are available to test publication bias, although none are perfect, are easily influenced by heterogeneity, and in our case two of them were used which gave inconsistent results. This fact illustrates that the role of existing publication bias cannot be completely ruled out. We cannot be sure whether to trust our funnel plots where most of the studies were contained within the inverted cone, signaling a lack of publication bias or the results of the Egger’s test where significant *p* values were seen for both genetic model comparisons in most of the analyzed groups/subgroups.

## Conclusions

Significant association of *IL6*-174 G/C variant with CAD was observed in the pooled results of our present meta-analysis, largely driven by studies belonging to Asian and Asian Indian ancestral subgroups. Upregulation of plasma IL-6 levels in the ‘C’ allele carriers seem to be at least partly responsible for this seen association. Further investigations are warranted with large structured case–control studies especially amongst populations belonging to Asian and Asian Indian ancestry.

## Supplementary Information

Below is the link to the electronic supplementary material.

Supplementary Figure 1. Forest plot depicting associations of *IL6* -174 G/C polymorphism with CAD employing a dominant genetic model (CC+GC vs. GG). Effect sizes for “Pooled” as well as for European and Asian Indian ancestral subgroups displaying a high level of heterogeneity were estimated using random effects for analysis. Effect sizes for Middle Eastern, Asian, African and Mixed ancestral subgroups were estimated using fixed effects. (PPTX 127 KB)

Supplementary Figure 2. Forest plot depicting associations of *IL6* -174 G/C polymorphism with CAD employing an allelic genetic model (Allele C vs. Allele G). Effect sizes for “Pooled” as well as for European and Asian Indian ancestral subgroups displaying high level heterogeneity were estimated using random effects for analysis. Effect sizes for Middle Eastern, Asian, African and Mixed ancestral subgroups were estimated using fixed effects. (PPTX 127 KB)

Supplementary Figure 3. Forest plots depicting differences in circulating IL-6 levels amongst ‘C’ allele carriers and GG homozygotes. Comparison of IL-6 levels in CC+GC vs. GG genotypes of *IL6* -174 G/C polymorphism separately amongst CAD cases and CAD free controls. Standard mean difference for “Pooled” as well as CAD case subgroup were estimated using random effects owing to high levels of inherent heterogeneity. Standard mean difference for CAD free control subgroup which displayed low levels of inherent heterogeneity were estimated using fixed effects. (PPTX 65 KB)

Supplementary Table 1 (DOCX 15 KB)

Supplementary Table 2 (DOCX 15 KB)
