# Supplementary material for: Association of interleukin 6 -174 G/C polymorphism with coronary artery disease and circulating IL-6 levels: a systematic review and meta-analysis
Source: Inflamm Res. 2021 Sep 30;70(10-12):1075–87. doi: 10.1007/s00011-021-01505-7 (PMC8572816; doi:10.1007/s00011-021-01505-7)
Supplement: Supplementary file 1 — Supplementary Figure 1. Forest plot depicting associations of IL6 -174 G/C polymorphism with CAD employing a dominant genetic model (CC+GC vs. GG). Effect sizes for “Pooled” as well as for European and Asian Indian ancestral subgroups displaying a high level of heterogeneity were estimated using random effects for analysis. Effect sizes for Middle Eastern, Asian, African and Mixed ancestral subgroups were estimated using fixed effects. (PPTX 127 KB) [file 11_2021_1505_MOESM1_ESM.pptx]

## Slide 1
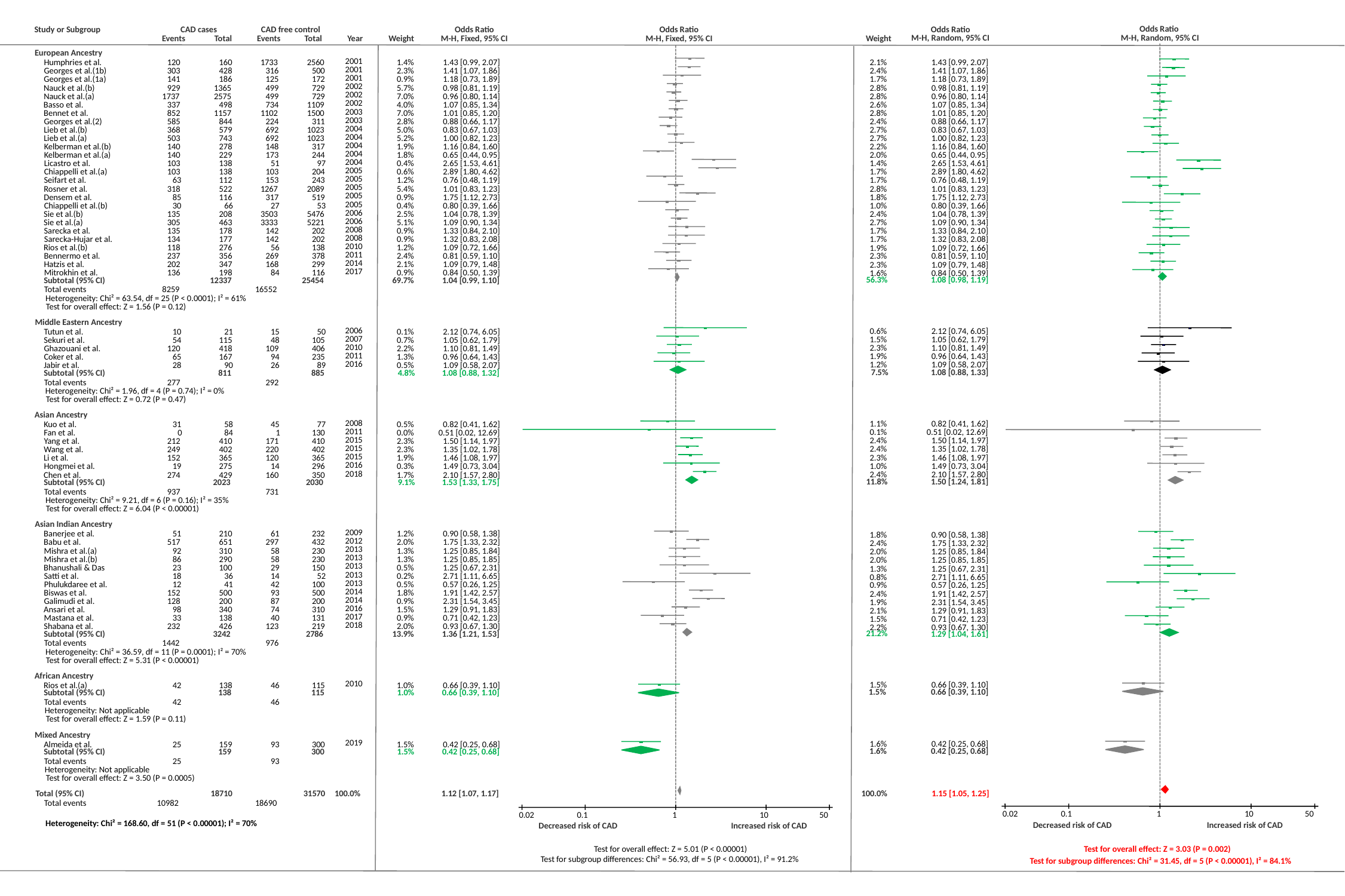

Odds Ratio
Odds Ratio
Study or Subgroup
CAD cases
CAD free control
Odds Ratio
Odds Ratio
M-H, Random, 95% CI
M-H, Random, 95% CI
Weight
Events
Total
Events
Total
Year
Weight
M-H, Fixed, 95% CI
M-H, Fixed, 95% CI
European Ancestry
2001
Humphries et al.
120
160
1733
2560
1.4%
1.43 [0.99, 2.07]
2.1%
1.43 [0.99, 2.07]
2001
Georges et al.(1b)
303
428
316
500
2.3%
1.41 [1.07, 1.86]
2.4%
1.41 [1.07, 1.86]
2001
Georges et al.(1a)
141
186
125
172
0.9%
1.18 [0.73, 1.89]
1.7%
1.18 [0.73, 1.89]
2002
Nauck et al.(b)
929
1365
499
729
5.7%
0.98 [0.81, 1.19]
2.8%
0.98 [0.81, 1.19]
2002
Nauck et al.(a)
1737
2575
499
729
7.0%
0.96 [0.80, 1.14]
2.8%
0.96 [0.80, 1.14]
2002
Basso et al.
337
498
734
1109
4.0%
1.07 [0.85, 1.34]
2.6%
1.07 [0.85, 1.34]
2003
Bennet et al.
852
1157
1102
1500
7.0%
1.01 [0.85, 1.20]
2.8%
1.01 [0.85, 1.20]
2003
2.4%
0.88 [0.66, 1.17]
Georges et al.(2)
585
844
224
311
2.8%
0.88 [0.66, 1.17]
2004
Lieb et al.(b)
368
579
692
1023
5.0%
0.83 [0.67, 1.03]
2.7%
0.83 [0.67, 1.03]
2004
Lieb et al.(a)
503
743
692
1023
5.2%
1.00 [0.82, 1.23]
2.7%
1.00 [0.82, 1.23]
2004
Kelberman et al.(b)
140
278
148
317
1.9%
1.16 [0.84, 1.60]
2.2%
1.16 [0.84, 1.60]
2004
Kelberman et al.(a)
140
229
173
244
1.8%
0.65 [0.44, 0.95]
2.0%
0.65 [0.44, 0.95]
2004
Licastro et al.
103
138
51
97
0.4%
2.65 [1.53, 4.61]
1.4%
2.65 [1.53, 4.61]
2005
Chiappelli et al.(a)
103
138
103
204
0.6%
2.89 [1.80, 4.62]
1.7%
2.89 [1.80, 4.62]
2005
1.7%
0.76 [0.48, 1.19]
Seifart et al.
63
112
153
243
1.2%
0.76 [0.48, 1.19]
2005
Rosner et al.
318
522
1267
2089
5.4%
1.01 [0.83, 1.23]
2.8%
1.01 [0.83, 1.23]
2005
Densem et al.
85
116
317
519
0.9%
1.75 [1.12, 2.73]
1.8%
1.75 [1.12, 2.73]
2005
Chiappelli et al.(b)
30
66
27
53
0.4%
0.80 [0.39, 1.66]
1.0%
0.80 [0.39, 1.66]
2006
Sie et al.(b)
135
208
3503
5476
2.5%
1.04 [0.78, 1.39]
2.4%
1.04 [0.78, 1.39]
2006
Sie et al.(a)
305
463
3333
5221
5.1%
1.09 [0.90, 1.34]
2.7%
1.09 [0.90, 1.34]
2008
Sarecka et al.
135
178
142
202
0.9%
1.33 [0.84, 2.10]
1.7%
1.33 [0.84, 2.10]
2008
Sarecka-Hujar et al.
134
177
142
202
0.9%
1.32 [0.83, 2.08]
1.7%
1.32 [0.83, 2.08]
2010
Rios et al.(b)
118
276
56
138
1.2%
1.09 [0.72, 1.66]
1.9%
1.09 [0.72, 1.66]
2011
Bennermo et al.
237
356
269
378
2.4%
0.81 [0.59, 1.10]
2.3%
0.81 [0.59, 1.10]
2014
Hatzis et al.
202
347
168
299
2.1%
1.09 [0.79, 1.48]
2.3%
1.09 [0.79, 1.48]
2017
Mitrokhin et al.
136
198
84
116
0.9%
0.84 [0.50, 1.39]
1.6%
0.84 [0.50, 1.39]
56.3%
1.08 [0.98, 1.19]
Subtotal (95% CI)
12337
25454
69.7%
1.04 [0.99, 1.10]
Total events
8259
16552
Heterogeneity: Chi² = 63.54, df = 25 (P < 0.0001); I² = 61%
Test for overall effect: Z = 1.56 (P = 0.12)
Middle Eastern Ancestry
2006
0.6%
2.12 [0.74, 6.05]
Tutun et al.
10
21
15
50
0.1%
2.12 [0.74, 6.05]
2007
1.5%
1.05 [0.62, 1.79]
Sekuri et al.
54
115
48
105
0.7%
1.05 [0.62, 1.79]
2010
2.3%
1.10 [0.81, 1.49]
Ghazouani et al.
120
418
109
406
2.2%
1.10 [0.81, 1.49]
2011
1.9%
0.96 [0.64, 1.43]
Coker et al.
65
167
94
235
1.3%
0.96 [0.64, 1.43]
2016
1.2%
1.09 [0.58, 2.07]
Jabir et al.
28
90
26
89
0.5%
1.09 [0.58, 2.07]
7.5%
1.08 [0.88, 1.33]
Subtotal (95% CI)
811
885
4.8%
1.08 [0.88, 1.32]
Total events
277
292
Heterogeneity: Chi² = 1.96, df = 4 (P = 0.74); I² = 0%
Test for overall effect: Z = 0.72 (P = 0.47)
Asian Ancestry
2008
1.1%
0.82 [0.41, 1.62]
Kuo et al.
31
58
45
77
0.5%
0.82 [0.41, 1.62]
2011
0.1%
0.51 [0.02, 12.69]
Fan et al.
0
84
1
130
0.0%
0.51 [0.02, 12.69]
2015
2.4%
1.50 [1.14, 1.97]
Yang et al.
212
410
171
410
2.3%
1.50 [1.14, 1.97]
2015
2.4%
1.35 [1.02, 1.78]
Wang et al.
249
402
220
402
2.3%
1.35 [1.02, 1.78]
2015
2.3%
1.46 [1.08, 1.97]
Li et al.
152
365
120
365
1.9%
1.46 [1.08, 1.97]
2016
1.0%
1.49 [0.73, 3.04]
Hongmei et al.
19
275
14
296
0.3%
1.49 [0.73, 3.04]
2018
2.4%
2.10 [1.57, 2.80]
Chen et al.
274
429
160
350
1.7%
2.10 [1.57, 2.80]
11.8%
1.50 [1.24, 1.81]
Subtotal (95% CI)
2023
2030
9.1%
1.53 [1.33, 1.75]
Total events
937
731
Heterogeneity: Chi² = 9.21, df = 6 (P = 0.16); I² = 35%
Test for overall effect: Z = 6.04 (P < 0.00001)
Asian Indian Ancestry
2009
Banerjee et al.
51
210
61
232
1.2%
0.90 [0.58, 1.38]
1.8%
0.90 [0.58, 1.38]
2012
Babu et al.
517
651
297
432
2.0%
1.75 [1.33, 2.32]
2.4%
1.75 [1.33, 2.32]
2013
Mishra et al.(a)
92
310
58
230
1.3%
1.25 [0.85, 1.84]
2.0%
1.25 [0.85, 1.84]
2013
Mishra et al.(b)
86
290
58
230
1.3%
1.25 [0.85, 1.85]
2.0%
1.25 [0.85, 1.85]
2013
Bhanushali & Das
23
100
29
150
0.5%
1.25 [0.67, 2.31]
1.3%
1.25 [0.67, 2.31]
2013
Satti et al.
18
36
14
52
0.2%
2.71 [1.11, 6.65]
0.8%
2.71 [1.11, 6.65]
2013
Phulukdaree et al.
12
41
42
100
0.5%
0.57 [0.26, 1.25]
0.9%
0.57 [0.26, 1.25]
2014
Biswas et al.
152
500
93
500
1.8%
1.91 [1.42, 2.57]
2.4%
1.91 [1.42, 2.57]
2014
Galimudi et al.
128
200
87
200
0.9%
2.31 [1.54, 3.45]
1.9%
2.31 [1.54, 3.45]
2016
Ansari et al.
98
340
74
310
1.5%
1.29 [0.91, 1.83]
2.1%
1.29 [0.91, 1.83]
2017
Mastana et al.
33
138
40
131
0.9%
0.71 [0.42, 1.23]
1.5%
0.71 [0.42, 1.23]
2018
Shabana et al.
232
426
123
219
2.0%
0.93 [0.67, 1.30]
2.2%
0.93 [0.67, 1.30]
21.2%
Subtotal (95% CI)
3242
2786
13.9%
1.36 [1.21, 1.53]
1.29 [1.04, 1.61]
Total events
1442
976
Heterogeneity: Chi² = 36.59, df = 11 (P = 0.0001); I² = 70%
Test for overall effect: Z = 5.31 (P < 0.00001)
African Ancestry
2010
1.5%
0.66 [0.39, 1.10]
Rios et al.(a)
42
138
46
115
1.0%
0.66 [0.39, 1.10]
1.5%
0.66 [0.39, 1.10]
Subtotal (95% CI)
138
115
1.0%
0.66 [0.39, 1.10]
Total events
42
46
Heterogeneity: Not applicable
Test for overall effect: Z = 1.59 (P = 0.11)
Mixed Ancestry
2019
1.6%
0.42 [0.25, 0.68]
Almeida et al.
25
159
93
300
1.5%
0.42 [0.25, 0.68]
1.6%
0.42 [0.25, 0.68]
Subtotal (95% CI)
159
300
1.5%
0.42 [0.25, 0.68]
Total events
25
93
Heterogeneity: Not applicable
Test for overall effect: Z = 3.50 (P = 0.0005)
100.0%
1.15 [1.05, 1.25]
Total (95% CI)
18710
31570
100.0%
1.12 [1.07, 1.17]
Total events
10982
18690
0.02
0.1
1
10
50
0.02
0.1
1
10
50
Heterogeneity: Chi² = 168.60, df = 51 (P < 0.00001); I² = 70%
Decreased risk of CAD
Increased risk of CAD
Decreased risk of CAD
Increased risk of CAD
Test for overall effect: Z = 5.01 (P < 0.00001)
Test for overall effect: Z = 3.03 (P = 0.002)
Test for subgroup differences: Chi² = 56.93, df = 5 (P < 0.00001), I² = 91.2%
Test for subgroup differences: Chi² = 31.45, df = 5 (P < 0.00001), I² = 84.1%
