# Supplementary material for: Association of interleukin 6 -174 G/C polymorphism with coronary artery disease and circulating IL-6 levels: a systematic review and meta-analysis
Source: Inflamm Res. 2021 Sep 30;70(10-12):1075–87. doi: 10.1007/s00011-021-01505-7 (PMC8572816; doi:10.1007/s00011-021-01505-7)
Supplement: Supplementary file 2 — Supplementary Figure 2. Forest plot depicting associations of IL6 -174 G/C polymorphism with CAD employing an allelic genetic model (Allele C vs. Allele G). Effect sizes for “Pooled” as well as for European and Asian Indian ancestral subgroups displaying high level heterogeneity were estimated using random effects for analysis. Effect sizes for Middle Eastern, Asian, African and Mixed ancestral subgroups were estimated using fixed effects. (PPTX 127 KB) [file 11_2021_1505_MOESM2_ESM.pptx]

## Slide 1
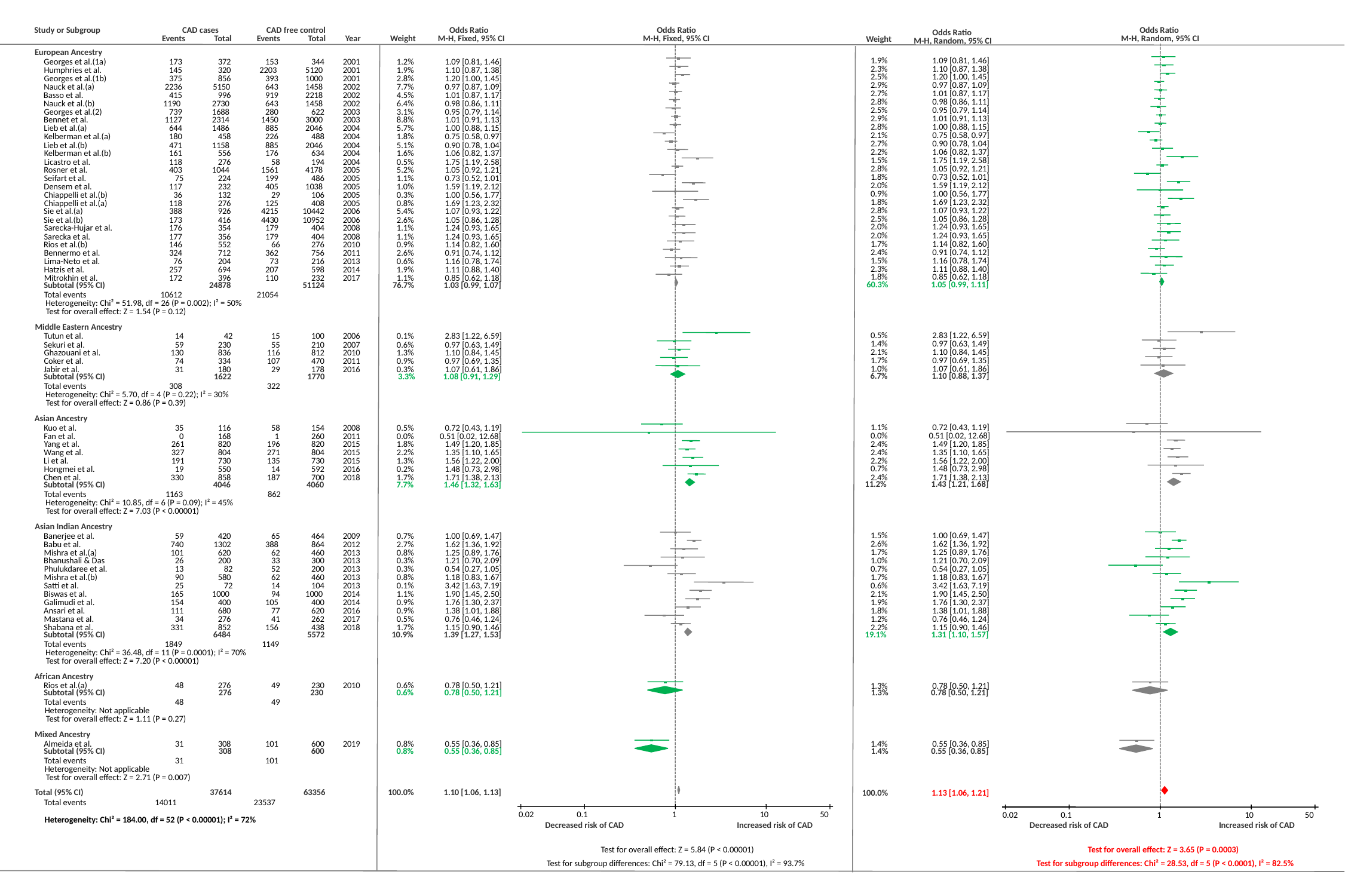

Study or Subgroup
CAD cases
CAD free control
Odds Ratio
Odds Ratio
Odds Ratio
Odds Ratio
Events
Total
Events
Total
Year
Weight
M-H, Fixed, 95% CI
M-H, Fixed, 95% CI
M-H, Random, 95% CI
Weight
M-H, Random, 95% CI
European Ancestry
1.9%
1.09 [0.81, 1.46]
Georges et al.(1a)
173
372
153
344
2001
1.2%
1.09 [0.81, 1.46]
2.3%
1.10 [0.87, 1.38]
Humphries et al.
145
320
2203
5120
2001
1.9%
1.10 [0.87, 1.38]
2.5%
1.20 [1.00, 1.45]
Georges et al.(1b)
375
856
393
1000
2001
2.8%
1.20 [1.00, 1.45]
2.9%
0.97 [0.87, 1.09]
Nauck et al.(a)
2236
5150
643
1458
2002
7.7%
0.97 [0.87, 1.09]
2.7%
1.01 [0.87, 1.17]
Basso et al.
415
996
919
2218
2002
4.5%
1.01 [0.87, 1.17]
2.8%
0.98 [0.86, 1.11]
Nauck et al.(b)
1190
2730
643
1458
2002
6.4%
0.98 [0.86, 1.11]
2.5%
0.95 [0.79, 1.14]
Georges et al.(2)
739
1688
280
622
2003
3.1%
0.95 [0.79, 1.14]
2.9%
1.01 [0.91, 1.13]
Bennet et al.
1127
2314
1450
3000
2003
8.8%
1.01 [0.91, 1.13]
2.8%
1.00 [0.88, 1.15]
Lieb et al.(a)
644
1486
885
2046
2004
5.7%
1.00 [0.88, 1.15]
2.1%
0.75 [0.58, 0.97]
Kelberman et al.(a)
180
458
226
488
2004
1.8%
0.75 [0.58, 0.97]
2.7%
0.90 [0.78, 1.04]
Lieb et al.(b)
471
1158
885
2046
2004
5.1%
0.90 [0.78, 1.04]
2.2%
1.06 [0.82, 1.37]
Kelberman et al.(b)
161
556
176
634
2004
1.6%
1.06 [0.82, 1.37]
1.5%
1.75 [1.19, 2.58]
Licastro et al.
118
276
58
194
2004
0.5%
1.75 [1.19, 2.58]
2.8%
1.05 [0.92, 1.21]
Rosner et al.
403
1044
1561
4178
2005
5.2%
1.05 [0.92, 1.21]
1.8%
0.73 [0.52, 1.01]
Seifart et al.
75
224
199
486
2005
1.1%
0.73 [0.52, 1.01]
2.0%
1.59 [1.19, 2.12]
Densem et al.
117
232
405
1038
2005
1.0%
1.59 [1.19, 2.12]
0.9%
1.00 [0.56, 1.77]
Chiappelli et al.(b)
36
132
29
106
2005
0.3%
1.00 [0.56, 1.77]
1.8%
1.69 [1.23, 2.32]
Chiappelli et al.(a)
118
276
125
408
2005
0.8%
1.69 [1.23, 2.32]
2.8%
1.07 [0.93, 1.22]
Sie et al.(a)
388
926
4215
10442
2006
5.4%
1.07 [0.93, 1.22]
2.5%
1.05 [0.86, 1.28]
Sie et al.(b)
173
416
4430
10952
2006
2.6%
1.05 [0.86, 1.28]
2.0%
1.24 [0.93, 1.65]
Sarecka-Hujar et al.
176
354
179
404
2008
1.1%
1.24 [0.93, 1.65]
2.0%
1.24 [0.93, 1.65]
Sarecka et al.
177
356
179
404
2008
1.1%
1.24 [0.93, 1.65]
1.7%
1.14 [0.82, 1.60]
Rios et al.(b)
146
552
66
276
2010
0.9%
1.14 [0.82, 1.60]
2.4%
0.91 [0.74, 1.12]
Bennermo et al.
324
712
362
756
2011
2.6%
0.91 [0.74, 1.12]
1.5%
1.16 [0.78, 1.74]
Lima-Neto et al.
76
204
73
216
2013
0.6%
1.16 [0.78, 1.74]
2.3%
1.11 [0.88, 1.40]
Hatzis et al.
257
694
207
598
2014
1.9%
1.11 [0.88, 1.40]
1.8%
0.85 [0.62, 1.18]
Mitrokhin et al.
172
396
110
232
2017
1.1%
0.85 [0.62, 1.18]
60.3%
1.05 [0.99, 1.11]
Subtotal (95% CI)
24878
51124
76.7%
1.03 [0.99, 1.07]
Total events
10612
21054
Heterogeneity: Chi² = 51.98, df = 26 (P = 0.002); I² = 50%
Test for overall effect: Z = 1.54 (P = 0.12)
Middle Eastern Ancestry
0.5%
2.83 [1.22, 6.59]
Tutun et al.
14
42
15
100
2006
0.1%
2.83 [1.22, 6.59]
1.4%
0.97 [0.63, 1.49]
Sekuri et al.
59
230
55
210
2007
0.6%
0.97 [0.63, 1.49]
2.1%
1.10 [0.84, 1.45]
Ghazouani et al.
130
836
116
812
2010
1.3%
1.10 [0.84, 1.45]
1.7%
0.97 [0.69, 1.35]
Coker et al.
74
334
107
470
2011
0.9%
0.97 [0.69, 1.35]
1.0%
1.07 [0.61, 1.86]
Jabir et al.
31
180
29
178
2016
0.3%
1.07 [0.61, 1.86]
6.7%
1.10 [0.88, 1.37]
Subtotal (95% CI)
1622
1770
3.3%
1.08 [0.91, 1.29]
Total events
308
322
Heterogeneity: Chi² = 5.70, df = 4 (P = 0.22); I² = 30%
Test for overall effect: Z = 0.86 (P = 0.39)
Asian Ancestry
1.1%
0.72 [0.43, 1.19]
Kuo et al.
35
116
58
154
2008
0.5%
0.72 [0.43, 1.19]
0.0%
0.51 [0.02, 12.68]
Fan et al.
0
168
1
260
2011
0.0%
0.51 [0.02, 12.68]
2.4%
1.49 [1.20, 1.85]
196
820
2015
1.8%
1.49 [1.20, 1.85]
Yang et al.
261
820
2.4%
1.35 [1.10, 1.65]
271
804
2015
2.2%
1.35 [1.10, 1.65]
Wang et al.
327
804
2.2%
1.56 [1.22, 2.00]
Li et al.
191
730
135
730
2015
1.3%
1.56 [1.22, 2.00]
0.7%
1.48 [0.73, 2.98]
Hongmei et al.
19
550
14
592
2016
0.2%
1.48 [0.73, 2.98]
2.4%
1.71 [1.38, 2.13]
187
700
2018
1.7%
1.71 [1.38, 2.13]
Chen et al.
330
858
11.2%
1.43 [1.21, 1.68]
4060
7.7%
1.46 [1.32, 1.63]
Subtotal (95% CI)
4046
Total events
1163
862
Heterogeneity: Chi² = 10.85, df = 6 (P = 0.09); I² = 45%
Test for overall effect: Z = 7.03 (P < 0.00001)
Asian Indian Ancestry
1.5%
1.00 [0.69, 1.47]
65
464
2009
0.7%
1.00 [0.69, 1.47]
Banerjee et al.
59
420
2.6%
1.62 [1.36, 1.92]
388
864
2012
2.7%
1.62 [1.36, 1.92]
Babu et al.
740
1302
1.7%
1.25 [0.89, 1.76]
Mishra et al.(a)
101
620
62
460
2013
0.8%
1.25 [0.89, 1.76]
1.0%
1.21 [0.70, 2.09]
Bhanushali & Das
26
200
33
300
2013
0.3%
1.21 [0.70, 2.09]
0.7%
0.54 [0.27, 1.05]
52
200
2013
0.3%
0.54 [0.27, 1.05]
Phulukdaree et al.
13
82
1.7%
1.18 [0.83, 1.67]
580
62
460
2013
0.8%
1.18 [0.83, 1.67]
Mishra et al.(b)
90
0.6%
3.42 [1.63, 7.19]
Satti et al.
25
72
14
104
2013
0.1%
3.42 [1.63, 7.19]
2.1%
1.90 [1.45, 2.50]
Biswas et al.
165
1000
94
1000
2014
1.1%
1.90 [1.45, 2.50]
400
105
400
2014
0.9%
1.76 [1.30, 2.37]
1.9%
Galimudi et al.
154
1.76 [1.30, 2.37]
1.8%
1.38 [1.01, 1.88]
680
77
620
2016
0.9%
1.38 [1.01, 1.88]
Ansari et al.
111
Mastana et al.
34
276
41
262
2017
0.5%
0.76 [0.46, 1.24]
1.2%
0.76 [0.46, 1.24]
2.2%
1.15 [0.90, 1.46]
Shabana et al.
331
852
156
438
2018
1.7%
1.15 [0.90, 1.46]
6484
5572
10.9%
1.39 [1.27, 1.53]
19.1%
Subtotal (95% CI)
1.31 [1.10, 1.57]
1149
Total events
1849
Heterogeneity: Chi² = 36.48, df = 11 (P = 0.0001); I² = 70%
Test for overall effect: Z = 7.20 (P < 0.00001)
African Ancestry
Rios et al.(a)
48
276
49
230
2010
0.6%
0.78 [0.50, 1.21]
1.3%
0.78 [0.50, 1.21]
1.3%
0.78 [0.50, 1.21]
276
230
0.6%
0.78 [0.50, 1.21]
Subtotal (95% CI)
49
Total events
48
Heterogeneity: Not applicable
Test for overall effect: Z = 1.11 (P = 0.27)
Mixed Ancestry
Almeida et al.
31
308
101
600
2019
0.8%
0.55 [0.36, 0.85]
1.4%
0.55 [0.36, 0.85]
Subtotal (95% CI)
308
600
0.8%
0.55 [0.36, 0.85]
1.4%
0.55 [0.36, 0.85]
101
Total events
31
Heterogeneity: Not applicable
Test for overall effect: Z = 2.71 (P = 0.007)
37614
63356
100.0%
1.10 [1.06, 1.13]
Total (95% CI)
100.0%
1.13 [1.06, 1.21]
Total events
14011
23537
0.02
0.1
1
10
50
0.02
0.1
1
10
50
Heterogeneity: Chi² = 184.00, df = 52 (P < 0.00001); I² = 72%
Decreased risk of CAD
Increased risk of CAD
Decreased risk of CAD
Increased risk of CAD
Test for overall effect: Z = 3.65 (P = 0.0003)
Test for overall effect: Z = 5.84 (P < 0.00001)
Test for subgroup differences: Chi² = 28.53, df = 5 (P < 0.0001), I² = 82.5%
Test for subgroup differences: Chi² = 79.13, df = 5 (P < 0.00001), I² = 93.7%
