# Supplementary material for: Association of interleukin 6 -174 G/C polymorphism with coronary artery disease and circulating IL-6 levels: a systematic review and meta-analysis
Source: Inflamm Res. 2021 Sep 30;70(10-12):1075–87. doi: 10.1007/s00011-021-01505-7 (PMC8572816; doi:10.1007/s00011-021-01505-7)
Supplement: Supplementary file 3 — Supplementary Figure 3. Forest plots depicting differences in circulating IL-6 levels amongst ‘C’ allele carriers and GG homozygotes. Comparison of IL-6 levels in CC+GC vs. GG genotypes of IL6 -174 G/C polymorphism separately amongst CAD cases and CAD free controls. Standard mean difference for “Pooled” as well as CAD case subgroup were estimated using random effects owing to high levels of inherent heterogeneity. Standard mean difference for CAD free control subgroup which displayed low levels of inherent heterogeneity were estimated using fixed effects. (PPTX 65 KB) [file 11_2021_1505_MOESM3_ESM.pptx]

## Slide 1
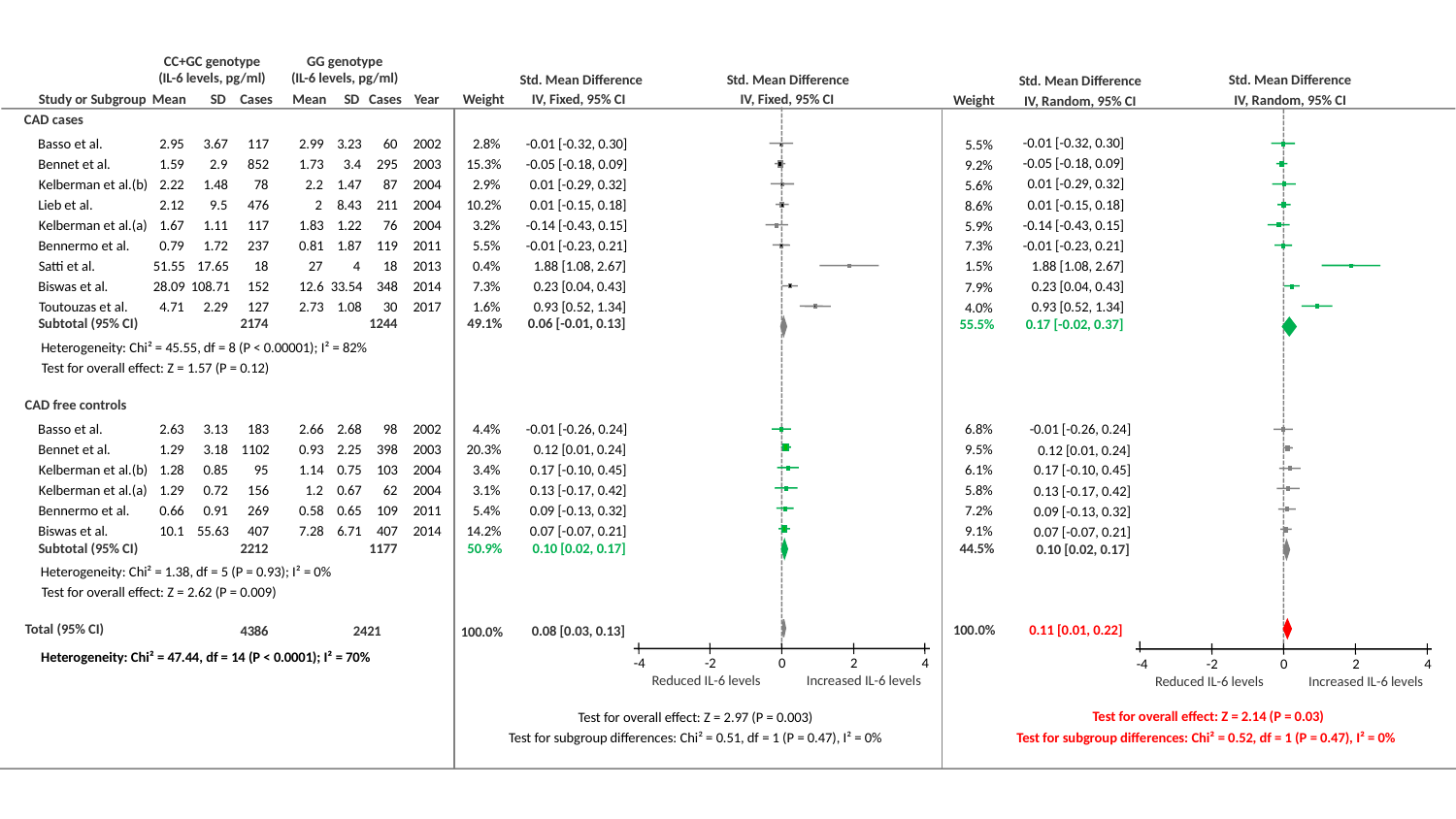

CC+GC genotype
(IL-6 levels, pg/ml)
GG genotype
(IL-6 levels, pg/ml)
Std. Mean Difference
Std. Mean Difference
Std. Mean Difference
Std. Mean Difference
Study or Subgroup
Mean
SD
Cases
Mean
SD
Cases
Year
Weight
IV, Fixed, 95% CI
IV, Fixed, 95% CI
Weight
IV, Random, 95% CI
IV, Random, 95% CI
CAD cases
-0.01 [-0.32, 0.30]
Basso et al.
2.95
3.67
117
2.99
3.23
60
2002
2.8%
-0.01 [-0.32, 0.30]
5.5%
-0.05 [-0.18, 0.09]
Bennet et al.
1.59
2.9
852
1.73
3.4
295
2003
15.3%
-0.05 [-0.18, 0.09]
9.2%
0.01 [-0.29, 0.32]
Kelberman et al.(b)
2.22
1.48
78
2.2
1.47
87
2004
2.9%
0.01 [-0.29, 0.32]
5.6%
Lieb et al.
2.12
9.5
476
2
8.43
211
2004
10.2%
0.01 [-0.15, 0.18]
0.01 [-0.15, 0.18]
8.6%
-0.14 [-0.43, 0.15]
Kelberman et al.(a)
1.67
1.11
117
1.83
1.22
76
2004
3.2%
-0.14 [-0.43, 0.15]
5.9%
Bennermo et al.
0.79
1.72
237
0.81
1.87
119
2011
5.5%
-0.01 [-0.23, 0.21]
-0.01 [-0.23, 0.21]
7.3%
1.88 [1.08, 2.67]
Satti et al.
51.55
17.65
18
27
4
18
2013
0.4%
1.88 [1.08, 2.67]
1.5%
Biswas et al.
28.09
108.71
152
12.6
33.54
348
2014
7.3%
0.23 [0.04, 0.43]
0.23 [0.04, 0.43]
7.9%
Toutouzas et al.
4.71
2.29
127
2.73
1.08
30
2017
1.6%
0.93 [0.52, 1.34]
0.93 [0.52, 1.34]
4.0%
Subtotal (95% CI)
2174
1244
49.1%
0.06 [-0.01, 0.13]
55.5%
0.17 [-0.02, 0.37]
Heterogeneity: Chi² = 45.55, df = 8 (P < 0.00001); I² = 82%
Test for overall effect: Z = 1.57 (P = 0.12)
CAD free controls
Basso et al.
2.63
3.13
183
2.66
2.68
98
2002
4.4%
-0.01 [-0.26, 0.24]
6.8%
-0.01 [-0.26, 0.24]
Bennet et al.
1.29
3.18
1102
0.93
2.25
398
2003
20.3%
0.12 [0.01, 0.24]
9.5%
0.12 [0.01, 0.24]
Kelberman et al.(b)
1.28
0.85
95
1.14
0.75
103
2004
3.4%
0.17 [-0.10, 0.45]
0.17 [-0.10, 0.45]
6.1%
Kelberman et al.(a)
1.29
0.72
156
1.2
0.67
62
2004
3.1%
0.13 [-0.17, 0.42]
5.8%
0.13 [-0.17, 0.42]
Bennermo et al.
0.66
0.91
269
0.58
0.65
109
2011
5.4%
0.09 [-0.13, 0.32]
7.2%
0.09 [-0.13, 0.32]
Biswas et al.
10.1
55.63
407
7.28
6.71
407
2014
14.2%
0.07 [-0.07, 0.21]
9.1%
0.07 [-0.07, 0.21]
Subtotal (95% CI)
2212
1177
50.9%
0.10 [0.02, 0.17]
44.5%
0.10 [0.02, 0.17]
Heterogeneity: Chi² = 1.38, df = 5 (P = 0.93); I² = 0%
Test for overall effect: Z = 2.62 (P = 0.009)
Total (95% CI)
0.11 [0.01, 0.22]
100.0%
4386
2421
0.08 [0.03, 0.13]
100.0%
Heterogeneity: Chi² = 47.44, df = 14 (P < 0.0001); I² = 70%
-4
-2
0
2
4
-4
-2
0
2
4
Reduced IL-6 levels
Increased IL-6 levels
Reduced IL-6 levels
Increased IL-6 levels
Test for overall effect: Z = 2.14 (P = 0.03)
Test for overall effect: Z = 2.97 (P = 0.003)
Test for subgroup differences: Chi² = 0.52, df = 1 (P = 0.47), I² = 0%
Test for subgroup differences: Chi² = 0.51, df = 1 (P = 0.47), I² = 0%
