# Supplementary material for: Association of interleukin 6 -174 G/C polymorphism with coronary artery disease and circulating IL-6 levels: a systematic review and meta-analysis
Source: Inflamm Res. 2021 Sep 30;70(10-12):1075–87. doi: 10.1007/s00011-021-01505-7 (PMC8572816; doi:10.1007/s00011-021-01505-7)
Supplement: Supplementary file 4 — Supplementary Table 1 (DOCX 15 KB) [file 11_2021_1505_MOESM4_ESM.docx]

**Supplementary Table 1. Sample sizes and heterogeneity in groups/sub-groups tested for CAD endpoint.**

|  | **Heterogeneity (*I^2^;* P_Q_)**  **[Number of studies included (**n_st_**) with number of case/controls assessed]** | | **Number of studies and sample assessed** [n_st_**; total sample (cases/controls)]** |
| --- | --- | --- | --- |
|  | ***Dominant genetic model^a^*** | ***Allelic genetic model^b^*** |  |
| Pooled | 70%; <0.00001  [n_st_= 52 (18,710/31,570)] | 72%; <0.00001  [n_st_= 53 (18,807/31,678)] | n_st_= 55; 51,213 (19,160/32,053) |
| European ancestry | 61%; <0.0001  [n_st_= 26 (12,337/25,454)] | 50%; 0.002  [n_st_= 27 (12,439/25,562)] | n_st_= 29; 38,724 (12,787/25,937) |
| Middle Eastern ancestry | 0%; 0.74  [n_st_= 5 (811/885)] | 30%; 0.22  [n_st_= 5 (811/885)] | n_st_= 5; 1,696 (811/885) |
| Asian ancestry | 35%; 0.16  [n_st_= 7 (2,023/2,030)] | 45%; 0.09  [n_st_= 7 (2,023/2,030)] | n_st_= 7; 4,053 (2,023/2,030) |
| Asian-Indian ancestry | 70%; 0.0001  [n_st_= 12 (3,242/2,786)] | 70%; 0.0001  [n_st_= 12 (3,242/2,786)] | n_st_= 12; 6,028 (3,242/2,786) |
| African ancestry | NA  [n_st_= 1 (138/115)] | NA  [n_st_= 1 (138/115)] | n_st_= 1; 253 (138/115) |
| Mixed Ancestry | NA  [n_st_= 1 (159/300)] | NA  [n_st_= 1 (159/300)] | n_st_= 1; 459 (159/300) |

Abbreviations- P_Q_: Cochran’s Q statistics; *I^2^*: Higgin’s *I^2^* statistics. *A derived P value of <0.05 was considered significant; **^R^**: Results derived using Random effects for analysis. Fixed effects were used for all other calculations; ^a^Dominant genetic model: CC+GC vs. GG; ^b^Allelic genetic model: Allele C vs. Allele G.
