# Supplementary material for: Association of interleukin 6 -174 G/C polymorphism with coronary artery disease and circulating IL-6 levels: a systematic review and meta-analysis
Source: Inflamm Res. 2021 Sep 30;70(10-12):1075–87. doi: 10.1007/s00011-021-01505-7 (PMC8572816; doi:10.1007/s00011-021-01505-7)
Supplement: Supplementary file 5 — Supplementary Table 2 (DOCX 15 KB) [file 11_2021_1505_MOESM5_ESM.docx]

**Supplementary Table 2. Meta-analysis results: Association of *IL6* -174 G/C polymorphism (rs1800795) with CAD.**

|  | **Dominant genetic model^a^** | | **Allelic genetic model^b^** | |
| --- | --- | --- | --- | --- |
|  | **OR, 95% CI** | **Z; P value** | **OR, 95% CI** | **Z; P value** |
| Pooled | 1.15, 1.05-1.25^R^ | 3.03; 0.002^R^* | 1.13, 1.06-1.21^R^ | 3.65; 0.0003^R^* |
| European ancestry | 1.08, 0.98-1.19^R^ | 1.58; 0.11^R^ | 1.05, 0.99-1.11^R^ | 1.60; 0.11^R^ |
| Middle Eastern ancestry | 1.08, 0.88-1.32 | 0.72; 0.47 | 1.08, 0.91-1.29 | 0.86; 0.39 |
| Asian ancestry | 1.53, 1.33-1.75 | 6.04; <0.0001* | 1.46, 1.32-1.63 | 7.03; <0.00001* |
| Asian Indian ancestry | 1.29, 1.04-1.61 | 2.28; 0.02^R^* | 1.31, 1.10-1.57^R^ | 2.95; 0.003^R^* |
| African ancestry | 0.66, 0.39-1.10 | 1.59; 0.11 | 0.78, 0.50-1.21 | 1.11; 0.27 |
| Mixed ancestry | 0.42, 0.25-0.68 | 3.50; 0.0005* | 0.55, 0.36-0.85 | 2.71; 0.007* |
| *Test for subgroup difference* | *I^2^*= 84.1%; P_Q_<0.00001^R^* | | *I^2^*= 82.5%; P_Q_<0.0001^R^* | |

Abbreviations- OR, 95% CI: Odds Ratio with its 95% Confidence Interval; P_Q_: Cochran’s Q statistics; *I^2^*: Higgin’s *I^2^* statistics.*A derived P value of <0.05 was considered significant; **^R^**: Results derived using Random effects for analysis. Fixed effects were used for all other calculations; ^a^Dominant genetic model: CC+GC vs. GG; ^b^Allelic genetic model: Allele C vs. Allele G.
